# Omalizumab in the treatment of eosinophilic granulomatosis with polyangiitis (EGPA): single-center experience in 18 cases

**DOI:** 10.1186/s40413-018-0217-0

**Published:** 2018-12-03

**Authors:** Zeynep Celebi Sozener, Begum Gorgulu, Dilsad Mungan, Betul Ayse Sin, Zeynep Misirligil, Omur Aydin, Sevim Bavbek

**Affiliations:** 1 Department of Chest Diseases, Division of Immunology and Allergy, School of Medicine, Ankara University, Ankara, Turkey; 2 Division of Allergy and Clinical Immunology Department of Chest Diseases, Ankara University School of Medicine, Ankara, Turkey

**Keywords:** EGPA, Churg-Strauss syndrome, Vasculitis, Asthma, Severe asthma, Omalizumab, Anti-IgE

## Abstract

**Background:**

Data are limited regarding the effectiveness of omalizumab in patients with eosinophilic granulomatosis with polyangiitis (EGPA). Our aim was to evaluate the clinical and functional effectiveness of omalizumab in patients with EGPA in long-term follow-up.

**Methods:**

This study was a retrospective chart review of patients with EGPA who were treated with omalizumab injections between May 2012 and April 2018. Once treatment with omalizumab was started, data were collected at various time points: baseline, the 16th week, 1st year, and annually until the last evaluation.

**Results:**

Eighteen patients (16F/2M) with a mean age of 48.61 ± 11.94 years were included. Data were available for all patients for the first year, 12 patients for the second year, 10 patients for the third  year, 8 patients for the fourth  year and 5 patients for the fifth year. All patients were on mean dosage of 15.77 ± 7.6 mg/day oral corticosteroid (OCS) as daily bases for mean 8.61 ± 4 years besides high-dose inhaler corticosteroid/long-acting beta agonist. Antineutrophil cytoplasmic antibodies (ANCA) were positive in 2  patients, and 8 patients were diagnosed as having vasculitis by skin biopsy, one patient had polyneuropathy, and one patient had cardiac involvement.

By considering the individual responses of patients and the level of improvement at the last evalulation, 10 (55.6%) patients responded completely, 1 responded partially, and 7 (38.9%) had no improvement. Omalizumab worked as a steroid-sparing agent in all patients and the daily OCS dose was reduced with a mean dosage of 6.28 mg/day at the end of the first year. The mean OCS reduction time for the whole group was 4 months. A reduction in asthma exacerbations/hospitalizations, improvement in forced expiratory volume in 1 second, and no decrease in the eosinophil count during treatment with omalizumab were also observed.

**Conclusions:**

Omalizumab improved asthma control in some patients with EGPA with uncontrolled asthma by reducing asthma exacerbations and oral steroid requirement. However, more data are needed before recommending widespread use of omalizumab in patients with EGPA.

## Background

Eosinophilic granulomatosis with polyangiitis (EGPA), formerly known as Churg–Strauss syndrome (CSS), is a rare systemic, small-to-medium vessel vasculitis associated with asthma, sinusitis, blood and tissue eosinophilia [1].

Conventional treatment of EGPA consisted of high doses of systemic steroids; however, for patients with severe or refractory diseases, immunosuppressive therapies including cyclophosphamide, azathioprine, intravenous immunglobulin (IVIG) are indicated [2]. Currently, an anti-interleukin (IL)-5 biologic agent, mepolizumab, has produced glucocorticoid reduction and protocol-defined remission in fifty perecent of patients with EGPA and has been approved by the United States Food and Drug Administration (FDA) in relapsing/refractory EGPA [2, 3].

Omalizumab, as an immunoglobulin (Ig)-E targeting biologic agent, has been demonstrated to be clearly effective in the treatment of patients with severe allergic asthma [4]. Omalizumab has also been reported to benefit patients with many different conditions such as allergic bronchopulmonary aspergillosis (ABPA), chronic urticaria, atopic dermatitis, and food allergy [5]. Besides blockage of free IgE, omalizumab reduces the recruitment and activation of eosinophils and other inflammatory cells to the inflammation site through the inhibition of Th2-type immune response [6, 7]. Considering the efficacy of omalizumab in the reduction of circulating and tissue eosinophils, the drug has also been given to patients with EGPA in off-label conditions, but experience is limited and even conflicting [8–10]. In the first reported case, a three-month administration of omalizumab to a patient with EGPA resulted in significant improvement of asthma and a marked decrease in the eosinophil count [8]. A recent multicenter study with seventeen patients with EGPA suggested that omalizumab might have a corticosteroid-sparing effect in patients with EGPA with asthma and/or sinusitis, but reducing the corticosteroid dose might also increase the risk of severe EGPA flares, which raises the question of the safety of omalizumab in patients with EGPA [11]. At an established allergy and clinical immunology referral center located in the capital city of Turkey, we have been using omalizumab since 2008 for patients with severe allergic asthma [12]. We recently reported our experience with omalizumab for patients with severe non-allergic asthma and ABPA [13, 14]. We have also prescribed the drug off-label in patients with EGPA whose asthma was uncontrolled despite receiving optimal treatment including long-term systemic steroids. Therefore, considering the limited experience in such cases, we aimed to evaluate the clinical and functional effectiveness of omalizumab in patients with EGPA in real-life settings.

## Methods

### Study design

**Fig. 1:**
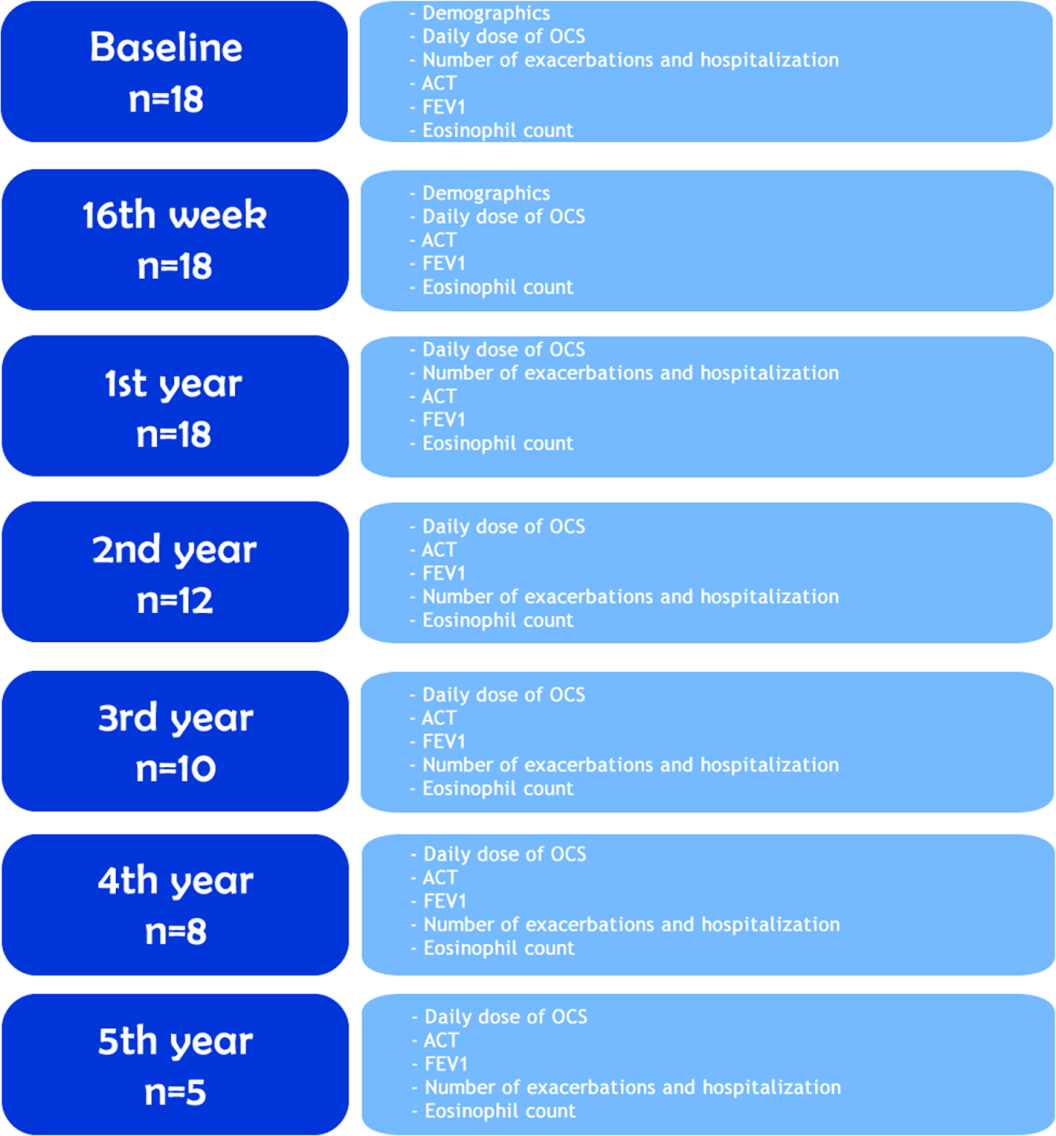
A flowchart of the patients at the time points

The study was conducted as a retrospective chart review of patients with EGPA who were treated with omalizumab between May 2012 and April 2018 in Ankara University, School of Medicine, Department of Chest Diseases, Division of Clinical Immunology and Allergy. The charts were reviewed by three physicians. Details of the evaluation, time points at which the assessments were conducted, and the number of patients are given in a flowchart (Fig. 1). The local ethics committee of Ankara University, School of Medicine, approved the study (Approval number: 07–453-18) and written informed consent was obtained from all subjects.

### Subjects

The diagnosis of asthma was made by using the clinical history and by demonstrating objective measures of reversible airway obstruction. In the diagnosis of EGPA, the criteria defined by the American College of Rheumatology (ACR) were used [15]. Accordingly, the diagnosis of EGPA is based on the presence of four or more of the following six findings at the time of the diagnosis: asthma, > 10% eosinophilia in a differential white blood cell count, mononeuropathy or polyneuropathy due to a systemic vasculitis, paranasal sinus abnormalities, migratory or transient pulmonary opacities, and histologic evidence of extravascular eosinophils in a biopsy specimen.

Patients with EGPA with at least 2 asthma exacerbations despite high-dose inhaled corticosteroid/long-acting beta agonist (ICS/LABA) treatment, and frequent or continuous oral corticosteroid (OCS) requirement with severe adverse effects were considered as candidates for omalizumab treatment. The omalizumab (Xolair, Genentech, Novartis, Sweden) dose was calculated from the chart according to the patients’ total IgE level and weight. Twelve patients received 150 mg omalizumab/month(m), 3 patients recieved 300 mg/m, 1 had 600 mg/m, one received 450 mg every 2 weeks (w), and 1 had 150 mg/2w; no dosing adjustments were made during the treatment period. Fourteen patients were still continuing omalizumab treatment and under regular follow-up in our department; 5 of whom use azathioprine as an add-on treatment. For 4 patients (patient # 2, 3, 14, 17) outside of these 14, omalizumab was discontiuned because of pregnancy (*n* = 1), gastric metaplasia (*n* = 2), and myalgia (*n* = 1), respectively.

### Measurements

Demographic features and clinical characteristics were recorded from the patients’ files. An asthma control test (ACT) and pulmonary function tests (PFT) (ZAN 100; nSpire, Oberthulba, Germany) (forced expiratory volume in 1 s [FEV1], forced vital capacity [FVC] and FEV1: FVC ratio) were routinely performed in all patients at every omalizumab visit. ICS doses were calculated as beclomethasone dipropionate (BDP), chlorofluorocarbon equivalent according to the Global Initiative for Asthma guidelines [16]. All patients were taking methylprednisolone as an OCS continuously and the doses given at the time points were calculated as a mean daily value.

### Outcomes

After treatment with omalizumab was started, data including PFTs, ACT scores, eosinophil count/percentage, and medications (OCS, ICS, LABA, and other controllers) for asthma were collected at baseline, the sixteenth week, first year, and annually thereafter. Outcome measurements are detailed in Fig. 1. The number of asthma exacerbations and hospitalizations for 1 year prior to omalizumab and yearly after starting omalizumab were also recorded.

The effectiveness of omalizumab treatment was determined according to asthma symptoms, decreases in mean daily OCS dosage and/or doses of ICS, LABA, and improvements in PFTs, decreases in asthma exacerbations, and in emergency visits and hospitalizations, which were assessed by at least 3 physicians who were all authors of this manuscript. Patients were classified as complete responders, partial responders, and refractory disease. Complete response was defined as the absence of asthma and/or ear, nose, and throat (ENT) exacerbations with a prednisone dosage of ≤7.5 mg/day, and partial response was defined as the absence of asthma and/or ENT exacerbations with a prednisone dosage of > 7.5 mg/day. Refractory disease was defined as the absence of improvement with omalizumab, i.e., presence of asthma and/or ENT exacerbations with a prednisone dosage of 7.5 mg/day. Relapsing disease was defined as initial improvement with complete response or partial response followed by disease flare. These definitions were formulated according to the recommendations of the EGPA Task Force and EULAR experts and approved for use in routine clinical practice [11].

### Statistical analysis

The statistical analysis was performed using SPSS version 20.0 (SPSS Inc., Chicago, IL, USA). Numeric values with normal dispersion are expressed as means±SD, and non-normally distributed variables are given as median values (min–max). Categorical variables are given as n (percentage). Time point comparisons were performed using repeated measures for variables with normal distribution and the Wilcoxon signed-rank test or Friedman’s test for variables with non-normal distribution. All directional *p* values were two-tailed and significance was assigned to values lower than 0.05.

## Results

### Demographics and diseases characteristics

Eighteen patients (16 women and 2 men) with a mean age of 48.61 ± 11.94 years were included. Patients were followed up regularly and had complete healthcare coverage. Data were available for all patients for the first year, 12 patients for the second year, 10 patients for the third year, eight patients for the fourth year, and 5  patients for the fifth year. The mean EGPA duration was 7.4 ± 4.3 years and the mean elapsed time between the diagnosis of asthma and diagnosis of EGPA was 8.5 ± 8.3 years. All patients were on high-dose ICS (min: 1000 μg BDP/day, max: 2000 μg, BDP/day) plus LABA and OCS. The baseline demographic findings and clinical characteristics of each subject in the study group are shown in Table 1. All patients in the study group used OCS as methylprednisolone at a mean dosage of 15.77 ± 7.6 mg/day (min: 4 mg/day, max: 60 mg/day) with a mean period of 8.61 ± 4 years (min: 4 years, max: 18 years). Nearly half of the patients were atopic (44.8%), 83.3% had sinusitis, and 33.3% had nasal polyposis. ANCA was only positive in 2 patients (patients #6, 11), and 8 patients (#3, 5, 9, 10, 12, 13, 14, 18) were diagnosed as having vasculitis via skin biopsy. Patient #15 had polyneuropathy and patient #17 had cardiac involvement.

**Table 1:** Demographic and disease characteristics

Patient/ sex/ Age	Duration of asthma (yr)	Duration of EGPA(yr)	Total IgE kU/L	Eosinophil cells/mm^3^, (%)	Radiologic Findings		Vasculitis	Polyeuropathy	Cardiac involvement	ANCA	Azathioprine	Omalizumab Dose (mg) / treatment duration (m)	Response
					Paranasal CT	Torax CT							
1.F/63	27	8	46.9	1700 (21.5)	Pansinusitis	Migratory ground glass opacities, bronchial wall thickness bronchiectasis	No	No	No	Neg	No	150/m/48 m	Complete
2.F/25	5	4	865	1800 (20.2)	Pansinusitis, nasal polyposis	Ground glass opacities, bronchial wall thickness	No	No	No	Neg	No	450/2w/15 m Stopped	None
3.F/52	24	21	11.8	13,480 (60)	Fronthoetmoid sinusitis, nasal polyposis	Ground glass opacities, bronchial wall thickness	Yes	No	No	Neg	No	150/m/18 m Stopped	None
4.F/44	11	8	47.9	3300 (27)	Pansinusitis	Transient ground glass opacities, bronchial wall thickness, milimetric nodules	No	No	No	Neg	No	150/m/44 m	Complete
5.F/47	12	4	134	1000 (15.9)	Pansinusitis	Ground glass opacities	Yes	No	No	Neg	No	300/m/39 m	Complete
6.F/67	22	8	17.8	8400 (53)	Pansinusitis	Transient ground glass opacities, milmetric nodules	No	Yes	No	Pos	No	150/m/62 m	Complete
7.F/62	19	4	114	1200 (20.3)	Pansinusitis	Transient ground glass opacities, bronchial wall thickness, mucous impactions	No	No	No	Neg	No	150/m/48 m	Complete
8.M/36	7	7	108	2900 (26)	Pansinusitis	Peribronchial thickness, ground glass opacities dominant at left upper lobe, focal patchy infiltrations	No	No	No	Neg	No	150/m/48 m	Complete
9.F/50	10	10	41	2500 (24.9)	Maxiller sinusitis, nasal poliposis	Mucous impaction, bronchial wall thickness, bronchiectasis	Yes	Yes	Yes	Neg	Yes	150/m/42 m	None
10.F/40	11	8	142	1100 (14)	Pansinusitis	Subpleural ground glass opacification, pulmonary nodules	Yes	No	No	Neg	Yes	150/m/60 m	Partial
11.F/52	19	3.5	543	13,600 (52.4)	Nasal poliposis	Bronchial wall thickness, sublobuler patchy infiltrations	No	No	No	Pos	No	600/m/27 m	None
12.M/38	17	11	126	8900 (47.5)	Pansinusitis	Paranchimal nodules, bronchial wall thickness	Yes	No	No	Neg	No	300/m/70 m	Complete
13.F/61	36	7	186	2700 (20.7)	Pansinusitis	Migratory ground glass opasities, nodules and mucus impaction	Yes	No	No	Neg	No	300/m/64 m	None
14.F/45	21	8	321	2200 (21.5)	Chronic sinusitis	Bilateral bronchial wall thickness	Yes	Yes	No	Neg	Yes	300/2w/14 m Stopped	None
15.F/46	22	4	46	3800 (28.8)	Frontoetmoid sinusitis, nasal polipozis	Transient ground glass opacities	No	Yes	No	Neg	No	150/m/18 m	Complete
16.F/37	9	5	87.5	2700 (36.5)	Pansinusitis, nasal polyposis	Transient ground glass opacities	No	No	No	Neg	Yes	150/m/20 m	None
17.F/50	12	12	29	5070 (35.9)	Pan sinusitis	Bronchial wall thickness	No	No	Yes	Neg	No	150/m/17 m Stopped	Complete
18.F/28	5	2	71	1440 (15.1)	Pansinusitis	Peribronchioler ground glass opasities, micronodules	Yes	No	No	Neg	Yes	150/m/18 m	Complete

The most frequently observed radiologic sign in patients with EGPA consisted of transient and often migratory ground-glass lung opacities [17], and these were seen in 72% (*n* = 13) of the patients in our group. Other relatively common findings were the presence of airway involvement consisting of bronchial dilatation, bronchial wall thickening, and small peribronchial and centrilobular nodules related to eosinophilic infiltration of the bronchial wall and asthma [17], and these were seen in 11% (*n* = 2), 55.5% (*n* = 10), and 33.3% (*n* = 6) of patients, respectively.

### Assessment of treatment response

**Fig. 2:**
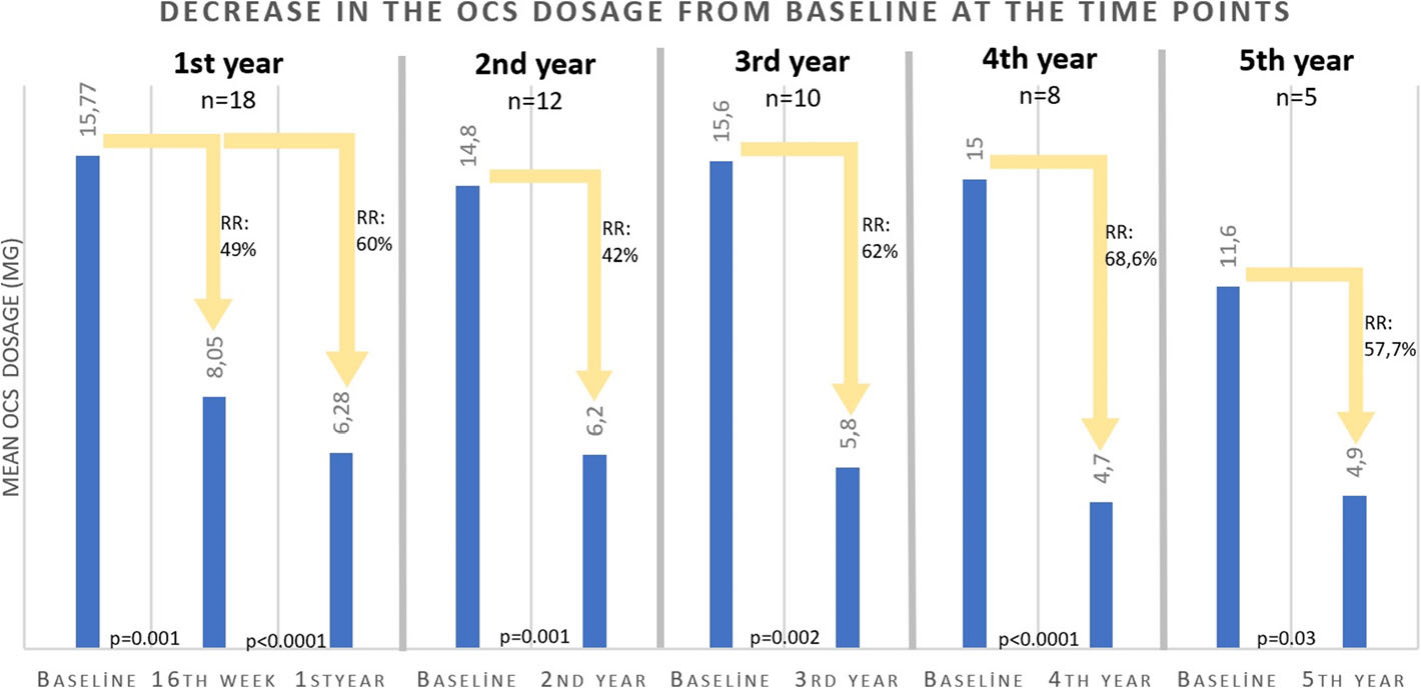
Decrease in the mean OCS dosage from baseline at the time points

**Fig. 3:**
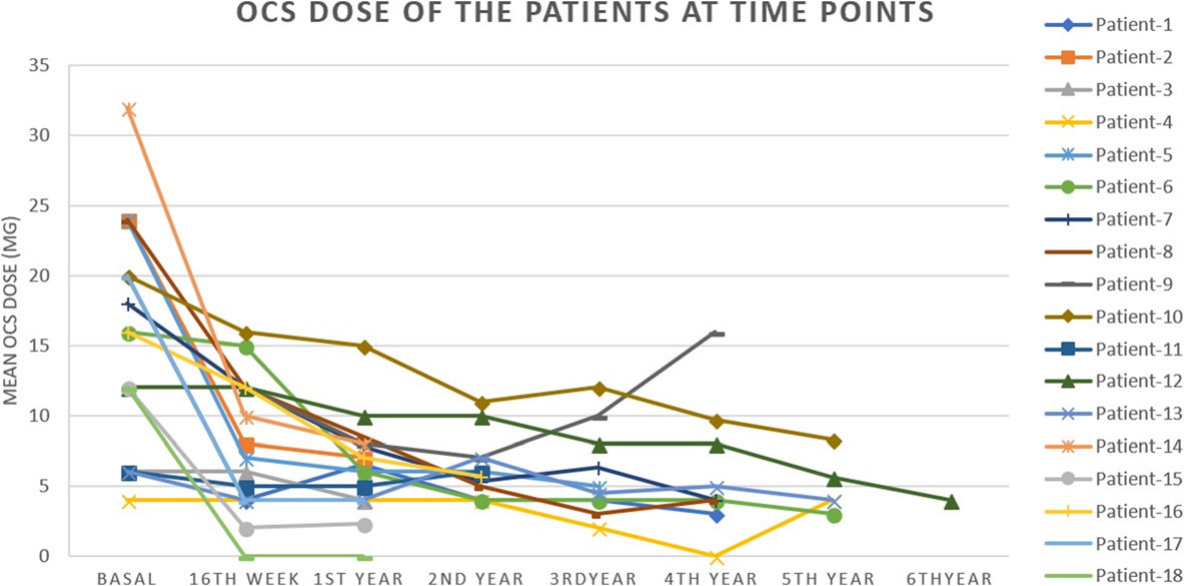
Mean OCS dosage of the patients at the time points

In general, considering the individual responses of the patients and the level of improvement at the last evalulation; 10 patients (55.6%) responded completely, one patient responded partially, and seven patients (38.9%) had no improvement. Approximately one-third of the patients received another immunosuppressive agent in addition to prednisone. The individual and the mean OCS dosage were significantly decreased at all time points and prednisone dosages were able to be tapered from a mean of 15.7 mg/day to 8.05 mg/day after 4 months (*p* = 0.001), to 6.28 mg/day at the first year (*p* < 0.0001) (*n* = 18). The dosages continued at this level for the second year of the study, *p* = 0.001 (*n* = 12), and then tapered again to 5.8 mg/day, 4.7 mg/day, and 4.9 mg/day at the end of the third *p* = 0.002 (*n* = 10), 4th *p* < 0.0001 (*n* = 8), and the fifth years *p* = 0.03 (*n* = 5) (Figs. 2 and 3). Only in one patient, OCS was discontinued after omalizumab therapy. The mean OCS reduction time for the entire group was 4 months (min 4, max 36 months).

The mean ACT score was increased at all timepoints compared with the baseline score (*p* = 0.03 for sixteenth week, *n* = 18; *p* = 0.07 for first year, *n* = 18; *p* = 0.05 for second year, *n* = 12; *p* = 0.15 for 3rd third year, *n* = 10; *p* = 0.01 for fourth year, *n* = 8; *p* = 0.06 for fifth year, *n* = 5).

**Fig. 4:**
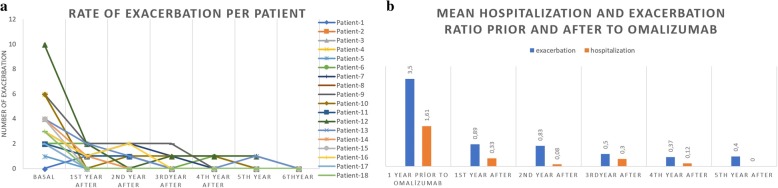
**a** The number of exacerbations per year for each patient **b** The mean exacerbation/hospitalization frequency per year, one year prior to omalizumab and annually thereafter

The baseline exacerbation rate was 3.56 ± 2.33 times per year (range, 1–10), and the hospitalization rate was 1.61 ± 1.72 times per year (range, 0–6) for 1 year prior to omalizumab, and both were significantly decreased at the first year (*n* = 18; *p* < 0.0001, *p* = 0.005), second year (*n* = 12; *p* = 0.01, *p* = 0.009), third year (*n* = 10; *p* = 0.006, *p* = 0.001), fourth year (*n* = 8; *p* = 0.03, *p* = 0.01), and fifth year (*n* = 5; *p* = 0.01, *p* = 0.04) (Fig. 4a, b).

According to the pulmonary function parameters, a significant increase in FEV1%, mL was observed only at the sixteenth week and first year vs. baseline in the entire group (p = 0.01, *p* = 0.08, *p* = 0.004, p = 0.01, respectively).

Total eosinophil numbers at the diagnosis of EGPA were higher in the non-responder group than in responder patients, although it did not reach statistical significance (5564 ± 5454 cells/mm^3^ vs. 3747 ± 2897 cells/mm^3^). Eosinophil numbers decreased among responders when we compared the data between the basal numbers of eosinophil and those of the first, second, and third year of follow-up (584 ± 304 cells/mm^3^, 508 ± 204 cells/mm^3^, 458 ± 194 cells/mm^3^, and 424 ± 93 cells/mm^3^, respectively). In contrast, there was a slight increase in eosinophil numbers in non-responders during the same follow-up period (395 ± 342 cells/mm^3^, 497 ± 214 cells/mm^3^, 412 ± 117 cells/mm^3^, and 667 ± 266 cells/mm^3^, respectively). We could not find a statistical significance either in increases or in decreases in the eosinophil counts within the groups because the numbers of patients were small.

Considering the individual responses of the patients: Patients # 1, 4, 5, 6, 7, 8, 12, 15, 17, and 18 were considered as complete responders in the last year and provided this response with under ≤7.5 mg prednisolone doses. Patient #1 had 2 exacerbations in the first 2 years and then no exacerbation in the following 2 years. Patients #6–7-12 also used ≤7.5 mg/day OCS dosages and had controlled disease without exacerbations or hospitalizations in last year of the omalizumab treatment but all had exacerbations and/or hospitalizations at some of the time points. Other patients had no exacerbations and hospitalizations after omalizumab was started and their ACT scores were increased at all time points. Patient #4’s OCS was stopped at the fourth year but was then started again at 4 mg/day dosage according to the elevation in eosinophil count. Patients #5, 12 and 18 had vasculitis. Patient #18’s OCS was stopped after omalizumab was started, but azathioprine was added becouse of vasculitis exacerbation in the first year of omalizumab treatment.

Patient #10 was considered a partial responder without exacerbation and hospitalization in the last year but they provided this response with under > 7.5 mg/day prednisolone dosage.

Patients #2, 3, 9, 11, 13, 14, and 16 were considered as non-responders. They all had exacerbations and/or hospitalizations (except patient #13) at all time points. In patient #13, disease control was provided nearly 2 years after beginning omalizumab treatment but she had 2 exacerbations in the fifth year of the therapy. Her OCS dose was changed between 7 and 4 mg/day dosages. These patients (except #2, 11, and 16), had vasculitis in the skin biopsies, and patient #11 had ANCA positivity. Patients #9 and 14 were under a high dosage of OCS (10 mg/day, 8 mg/day, respectively) and add-on azathioprine treatment. Patient 16 also took azathioprine as add-on to a 5.7 mg/day OCS dosage. Patient #2 had one exacerbation under a 7 mg/day prednisolone dosage but omalizumab was not continued because of pregnancy at the end of 15 months. Patient #3 had two exacerbations and one hospitalization under 4 mg/day OCS but omalizumab was not continued because of gastric metaplasia.

In the comparison of responders and non-responders, patients with complete response had shorter asthma and EGPA disease duration but had been taking omalizumab for longer than the non-responder patients (15.4 ± 7.1 vs. 17.71 ± 10.6 years; 6.8 ± 3.2 vs. 8.3 ± 6 years; 42.6 ± 19 vs. 28.57 ± 18.37 months, respectively). They had lower C-reactive protein (CRP) and sedimentation levels and lower eosinophil percentages than the patients with no response (2.2 ± 3.2 vs. 5.1 ± 5.9; 12.3 ± 7 vs. 19.7 ± 10; 29.1 ± 12.7% vs. 33 ± 16% respectively). Moreover, non-responder patients were using OCS for longer than the patients with complete response (10.4 ± 5.4 years vs. 7.3 ± 2.4 years). In non-responders, the number of patients diagnosed as having vasculitis was higher than among complete responders [4/7 (57%), 3/10 (30%), respectively]; all these data were statistically insignificant.

Except for 3 cases, omalizumab was well tolerated and no serious adverse events were observed. In patient #3 and patient #14, omalizumab was discontinued because of gastric metaplasia at 18 and 15 months after initiation, respectively. Patient #17 developed myalgia; EGPA involvement was excluded after neurologic and electromyographic examination, thus this was accepted as an adverse event and omalizumab was stopped at 17 months after initiation.

## Discussion

Our case series demonstrated that treatment with omalizumab in some patients with EGPA was effective in improving asthma symptoms and reducing OCS requirement, along with reducing asthma exacerbations and hospitalizations. There was also an improvement in functional parameters measured using FEV_1_. Therapeutic response appeared to be independent of asthma and EGPA diseases duration, duration of omalizumab OCS use, having vasculitis, CRP levels, sedimentation levels, and eosinophil numbers, but we could not analyze which patient characteristics would predict omalizumab responsiveness due to the small number of patients.

EGPA is presently defined as a syndrome consisting of 3 components; hypereosinophilic syndrome, ANCA-associated vasculitic manifestations, and asthma/ rhinosinusitis. Eosinophils are abundant and sustained both in the blood and in tissue and possibly play a central and/or additional role in the development of EGPA. Asthma and rhinosinusitis are the main features of the disease and almost all patients with EGPA have a history of nasal involvement and late-onset asthma [2, 18, 19]. As it was pointed out in the current study, the 3 components of EGPA may require separate approaches for their management because persistent eosinophilic inflammation in the upper and lower airways has been documented in patients with EGPA, although they were receiving low-dose oral corticosteroids and immunomodulating drugs and were in remission from systemic manifestations of the disease [1, 20].

Given the antiallergic and anti-inflammatory effectiveness of omalizumab, including the reduction of circulating and tissue eosinophils, it was proposed that omalizumab could be used to decrease eosinophilic activity resulting in asthma control in patients with EGPA [6, 7, 18]. Hovewer, there are limited data in single case reports/case series regarding the efficacy of omalizumab in patients with EGPA [8, 9, 21–23]. In the first case, the same group presented 3 months’ and 1 years’ administration of omalizumab. The patient showed significant improvement of asthma symptoms and a marked improvement in eosinophilia in both time periods [8, 22, 23]. Later, in the first documented pediatric case with EGPA, omalizumab treatment was demonstrated to control asthma as well as gastrointestinal symptoms [9]. There are recent data from a multicenter retrospective study including 17 patients with EGPA who received omalizumab as adjunctive therapy for refractory and/or relapsing asthmatic and/or sinonasal manifestations. After a median follow-up of 22 months, 6 (35%) patients achieved a complete response, 5 patients (30%) achieved a partial response, and 6 patients (35%) had no improvement based on the defined response criteria [11]. The median prednisone dosage decreased from 16 mg/day to 10 mg/day after 3 months of therapy and this reduction continued during the 12 months of follow-up whereas no significant difference was noted for the eosinophil count. Similarly, the number of asthma exacerbations also decreased, and the FEV_1_ value increased. The authors concluded that in EGPA patients with asthmatic and/or sinonasal manifestations, omalizumab had mild efficacy for the treatment of asthma and/or ENT symptoms [11]. Our data were quite similar to the results reported in that study in terms of responder and non-responder rates, reductions in mean dosage of daily OCS, reductions in asthma exacerbations, improvements in FEV1, and no decrease in eosinophil counts in whole group. However, we observed a tendency for a decrease in blood eosinophil numbers among omalizumab responder patients. In contrast, there was a slight increase in eosinophil numbers in non-responders during the same follow-up period. The responsiveness to omalizumab might be related to the reduction in the numbers of eosinophils, and non-responsiveness may be related with the lack of efficacy of omalizumab in the extensive eosinophilic infiltration derived from the Th2 response and different sources (1). However, we could not found a statistical significance either in increases or in decreases in the eosinophil counts within the groups. The limited number of patients prevents us from speculating on that issue. The immunopathogenesis of EGPA is still poorly understood, and large and long-term studies would be appropriate to determine the mechanistic role of omalizumab in patients with EGPA.

Systemic steroids are the cornerstone of EGPA treatment. They are combined with immune-suppressants such as cyclophosphamide and azathioprine, when necessary, as steroid-sparing agents. Persistent asthma is a major problem for patients with EGPA and exacerbations can occur repeatedly throughout the disease course, especially when the prednisone dosage is lower than 10 mg/day, thus leading to the concern for steroid-associated adverse reactions [1, 2, 19, 24–26]. A glucocorticoid-sparing effect of omalizumab has been reported in patients with severe allergic asthma [4], patients with nonallergic asthma, and ABPA [5, 13, 14]. In our trial, all patients were on mean dosage of 15.77 ± 7.6 mg methylprednisolone as daily bases for a mean 8.61 ± 4 years besides high-dose ICS-LABA and other controller medications for the management of EGPA. They had osteoporosis, obesity, diabetes mellitus, and steroid acnes as common steroid-induced adverse effects. As reported previously [11], omalizumab seemed to work as a steroid-sparing agent in all patients in our group, and the daily OCS dosage was reduced to 6.28 mg/day at the end of the first year. This decrease in the prednisolone dosage is a substantial clinical benefit when the adverse effects of long-term steroid treatment are considered. In 4 out of 8 patients with vasculitis, vasculitis flared when the steroid dosage was reduced. Azathioprine was added to omalizumab in only 5 patients as a glucocorticoid-sparing agent or treatment of cutaneous vasculitis.

The diagnosis of EGPA can be challenging because there is a lack of agreement on the diagnostic criteria, which usually relies on the characteristics of clinical manifestations and ANCA when present. We used the diagnostic criteria of ACR, the most commonly used criteria because they have been used in previous studies evaluating the effect of omalizumab in EGPA [8, 9, 11, 15]. There may be 2 EGPA phenotypes depending on the presence or absence of ANCA: The vasculitic and ANCA-positive phenotype, characterized by small vessel vasculitic features such as purpura, mono neuritis multiplex, glomerulonephritis, and the eosinophilic, ANCA-negative phenotype, characterized by peripheral eosinophilia and eosinophilic tissue infiltration (e.g., pulmonary infiltrates, cardiomyopathy) [2, 17, 19, 24, 27–29]. Our study group mostly comprised ANCA-negative patients, with only 2 ANCA-positive subjects (#6, 11). One of these ANCA-positive patients also had polyneuropathy in addition to pulmonary involvement. All ANCA-negative patients had pulmonary involvement and half had cutaneusvasculitis in the skin biopsy, 3 had polyneuropathy and two had cardiac involvement. Skin was the second most common organ involved in the EGPA presentation in our group. EGPA which is ANCA positive is predominantly in vasculitic from whereas eosinophilic tissue involvement is predominant in ANCA negative ones. Confirming this with future studies may provide inddividualization of treatment [24, 28, 30]. In our series, none of the patients with vasculitis had ANCA positivity; however, the number of subjects in our study group was limited.

Clinically, 3 distinct phases in the development of EGPA have been shown. These include (a) the prodromal period, which may persist for several years, consisting of asthma and allergic rhinitis; (b) a phase of marked peripheral blood eosinophilia and eosinophil tissue infiltrates; (c) and a life-threatening vasculitic phase [2, 31]. The first 2 prevasculitic phases are characterized by marked tissue eosinophilia, which manifests in the lung as eosinophilic pneumonia [32]. In our study, 8 patients had vasculitis shown by skin biopsy.The remaining patients were in the pre-vasculitic phases. Accordingly, migratory infiltration indicating pulmonary eosinophilic involvement was demonstrated using high-resolution computed tomography (HRCT) in all patients, along with increased peripheral eosinophilia, suggesting that most of our patients were in pre vasculitic phase.

The study had limitations such as the retrospective nature and the small number of subjects without a control group. However, there are only case reports and case series reported and only one multicenter study with 17 patients with EGPA and no controls [11]. To the best of our knowledge, as a single center, we had the largest number of patients with EGPA. Furthermore, off-label use of omalizumab in such patients is a limiting factor for conducting studies with larger numbers of patients. We used the patients’ own data before treatment with omalizumab as a comparison. However, incomplete data may have been obtained regarding doses of OCS due to self-managing dosing by patients and/or concomitant primary care prescribing and further supplies from admissions at other hospitals and/or emergency units. For the pre-omalizumab course, we made assumptions regarding the typical use of steroids for exacerbations in our country, which starts with a 40 mg dose of methyl prednisolone and the dose is then tapered to 8 mg at 3-day intervals for 15 days, when we could not obtain clear information. We believe that we had more accurate data about the doses of OCS because the patients were monitored more closely during the post-omalizumab period.

On the other hand, the study had some advantages such as the long follow-up period, which had a mean of 39 months. The longest follow up in the literature was a median 22 months in the multicenter study [11]. For the remaining cases series, data were reported for 3–12 months of treatment with omalizumab [8–10, 33]. Therefore, we believe that it is important to see the long-term effects of omalizumab in this disease, which has complex underlying immunopathogenesis.

## Conclusions

EGPA is a heterogeneous disease with several endotypes. This study indicates that omalizumab improved asthma control in some patients with EGPA with uncontrolled asthma by reducing asthma exacerbations and doses of oral steroids. However, more data are needed before recommending the use of omalizumab in patients with EGPA.

## Abbreviations

ABPA: Allergic bronchopulmonary aspergillosis
ACR: American Collage of Rheumatology
ACT: Asthma control test
ANCA: Antineutrophil cytoplasmic antibodies
BDP: Beclomethasone dipropionate
CRP: C-reactive protein
CSS: Churg–Strauss syndrome
EGPA: Eosinophilic granulomatosis with polyangiitis
ENT: Ear, nose, and throat
FDA: Food and Drug Administration EULAR: European League Against Rheumatism
FEV1: Forced expiratory volume in 1 s
FVC: Forced vital capacity
HRCT: High-resolution computed tomography
ICS/LABA: Inhaled corticosteroid/long-acting beta agonist
Ig: Immunglobulin
IVIG: Intravenous immunglobulin
m: Month
OCS: Oral corticosteroid
PFT: pulmonary function tests
w: Week

## References

[1] Raffray L, Guillevin L (2018). Treatment with eosinophilic granulomatosis with polyangiitis: a review. Drugs.

[2] Greco A, Rizzo MI, De Virgilio A, Gallo A, Fusconi M, Ruoppolo G, Altissimi G, De Vincentiis M (2015). Churg-Strauss syndrome. Autoimmun Rev.

[3] Wechsler ME, Akuthota P, Jayne D, Khoury P, Klion A, Langford CA, Merkel PA, Moosig F, Specks U, Cid MC, Luqmani R, Brown J, Mallett S, Philipson R, Yancey SW, Steinfeld J, Weller PF, Gleich GJ (2017). EGPA Mepolizumab study team. Mepolizumab or placebo for eosinophilic granulomatosis with Polyangiitis. N Engl J Med.

[4] Humbert M, Beasley R, Ayres J (2005). Benefits of omalizumab as add-on therapy in patients with severe persistent asthma who are inadequately controlled despite best available therapy (GINA 2002 step 4 treatment): INNOVATE. Allergy.

[5] Stokes JR, Casal TB (2015). The use of anti-IgE therapy beyond allergic asthma. J Allergy Clin Immunol Pract.

[6] Noga O, Hanf G, Brachmann I, Klucken AC, Kleine-Tebbe J, Rosseau S (2006). Effect of omalizumab treatment on peripheral eosinophil and T-lymphocyte function in patients with allergic asthma. J Allergy Clin Immunol.

[7] Djukanovic R, Wilson SJ, Kraft M, Jarjour NN, Steel M, Chung KF (2004). Effects of treatment with anti-immunoglobulin E antibody omalizumab on airway inflammation in allergic asthma. Am J Resp Crit Care Med.

[8] Giavina-Bianchi P, Giavina-Bianchi M, Agondi R, Kalil J (2007). Three months’ administration of anti-IgE to a patient with Churg-Strauss syndrome. J Allergy Clin Immunol.

[9] Iglesias E, Camacho Lovillo M, Delgado Pecellın I, Lirola Cruz MJ, Falcon Neyra MD, Salazar Quero JC (2014). Successful management of Churg-Strauss syndrome using omalizumab as adjuvant immunomodulatory therapy: first documented pediatric case. Pediatr Pulmonol.

[10] Ruppert AM, Averous G, Stanciu D, Deroide N, Riehm S, Poindron V (2008). Development of Churg-Strauss syndrome with controlled asthma during omalizumab treatment. J Allergy Clin Immunol.

[11] Jachiet M, Samson M, Cottin V, Kahn JE, Le Guenno G, Bonniaud P, Devilliers H, Bouillet L, Gondouin A, Makhlouf F, Meaux-Ruault N, Gil H, Bienvenu B, Coste A, Groh M, Giraud V, Dominique S, Godeau B, Puéchal X, Khouatra C, Ruivard M, Le Jeunne C, Mouthon L, Guillevin L, Terrier B (2016). French Vasculitis study group. Anti-IgE monoclonal antibody (Omalizumab) in refractory and relapsing eosinophilic granulomatosis with Polyangiitis (Churg-Strauss): data on seventeen patients. Arthritis Rheumatol.

[12] Bavbek S, Aydın O, Kepil Özdemir S, Yılmaz I, Celik GE, Demirel YS, Mungan D, Sin B, Kurşun N, Mısırlıgil Z (2010). Therapy with omalizumab in patients with severe persistent allergic asthma: a reallife data in Turkey. Tuberk Toraks.

[13] Celebi Sözener Z, Aydın Ö, Mısırlıgil Z, Mungan D, Demirel YS, Çelik GE, Sin BA, Bavbek S (2017). Omalizumab in non-allergic asthma: a report of 13 cases. J Asthma.

[14] Aydın Ö, Sözener ZÇ, Soyyiğit Ş, Kendirlinan R, Gençtürk Z, Mısırlıgil Z, Mungan D, Sin BA, Demirel YS, Çelik GE, Bavbek S (2015). Omalizumab in the treatment of allergic bronchopulmonary aspergillosis: one center’s experience with 14 cases. Allergy Asthma Proc.

[15] Masi AT, Hunder GG, Lie JT (1990). The American College of Rheumatology 1990 criteria for the classification of Churg-Strauss syndrome (allergic granulomatosis and angiitis). Arthritis Rheum.

[16] Global Initiative for Asthma (GINA) (2018). *Global* Strategy *for Asthma Management and* Prevention.

[17] Feragalli B, Mantini C, Sperandeo M, Galluzzo M, Belcaro G, Tartaro A (2016). The lung in systemic vasculitis: radiological patterns and differential diagnosis. Br J Radiol.

[18] Seo P (2016). Eosinophilic granulomatosis with Polyangiitis: challenges and opportunities. J Allergy Clin Immunol Pract.

[19] Pagnoux C (2016). Updates in ANCA-associated vasculitis. Eur J Rheumatol.

[20] Latorre M, Baldini C, Seccia V, Pepe P, Novelli F, Celi A, Bacci E, Cianchetti S, Dente FL, Bombardieri S, Paggiaro P (2016). Asthma control and airway inflammation in patients with eosinophilic granulomatosis with Polyangiitis. J Allergy Clin Immunol Pract.

[21] Spina MF, Miadonna A (2009). Role of omalizumab and steroids in Churg-Strauss syndrome. J Allergy Clin Immunol.

[22] Giavina-Bianchi P, Giavina-Bianchi M, Agondi R, Kalil J (2007). Administration of anti-IgE to a Churg-Strauss syndrome patient. Int Arch Allergy Immunol.

[23] Giavina-Bianchi P, Agondi R, Kalil J (2008). One year administration of anti-IgE to a Churg-Strauss syndrome patient. Int Arch Allergy Immunol.

[24] Pagnoux C, Guilpain P, Guillevin L (2007). Churg-Strauss syndrome. Curr Opin Rheumatol.

[25] Groh M, Pagnoux C, Guillevin L (2015). Eosinophilic granulomatosis with polyangiitis (formerly Churg-Strauss syndrome): where are we now?. Eur Respir J.

[26] Groh M, Pagnoux C, Baldini C, Bel E, Bottero P, Cottin V, Dalhoff K, Dunogué B, Gross W, Holle J, Humbert M, Jayne D, Jennette JC, Lazor R, Mahr A, Merkel PA, Mouthon L, Sinico RA, Specks U, Vaglio A, Wechsler ME, Cordier JF, Guillevin L (2015). Eosinophilic granulomatosis with polyangiitis (Churg-Strauss) (EGPA) consensus task force recommendations for evaluation and management. Eur J Intern Med.

[27] Gioffredi A, Maritati F, Oliva E, Buzio C (2014). Eosinophilic granulomatosis with polyangiitis: an overview. Front Immunol.

[28] Pagnoux C, Guillevin L (2010). Churg-Strauss syndrome: evidence for disease subtypes?. Curr Opin Rheumatol.

[29] Jennette JC (2013). Overview of the 2012 revised international Chapel Hill consensus conference nomenclature of vasculitides. Clin Exp Nephrol.

[30] Dunogue B, Pagnoux C, Guillevin L (2011). Churg-Strauss syndrome: clinical symptoms, complementary investigations, prognosis and utcome, and treatment. Semin Respir Crit Care Med.

[31] Kim YK, Lee KS, Chung MP, Han J, Chong S, Chung MJ (2007). Pulmonary involvement in Churg–Strauss syndrome: an analysis of CT, clinical, and pathologic findings. Eur Radiol.

[32] Silva CI (2005). M¨uller NL, Fujimoto K, Johkoh T, Ajzen SA, Churg a. Churg–Strauss syndrome: high resolution CT and pathologic findings. J Thorac Imaging.

[33] Cazzola M, Mura M, Segreti A, Mattei MA, Rogliani P (2009). Eosinophilic pneumonia in an asthmatic patient treated with omalizumab therapy: forme-fruste of Churg-Strauss syndrome?. Allergy.

